# Design of a *k*-space spectrometer for ultra-broad waveband spectral domain optical coherence tomography

**DOI:** 10.1038/srep42353

**Published:** 2017-03-07

**Authors:** Gongpu Lan, Guoqiang Li

**Affiliations:** 1 Visual and Biomedical Optics Lab, The Ohio State University, Columbus, OH 43212, USA; 2 Department of Ophthalmology and Visual Science, The Ohio State University, Columbus, OH 43212, USA; 3 Department of Electrical and Computer Engineering, The Ohio State University, Columbus, OH 43212, USA

## Abstract

Nonlinear sampling of the interferograms in wavenumber (*k*) space degrades the depth-dependent signal sensitivity in conventional spectral domain optical coherence tomography (SD-OCT). Here we report a linear-in-wavenumber (*k*-space) spectrometer for an ultra-broad bandwidth (760 nm–920 nm) SD-OCT, whereby a combination of a grating and a prism serves as the dispersion group. Quantitative ray tracing is applied to optimize the linearity and minimize the optical path differences for the dispersed wavenumbers. Zemax simulation is used to fit the point spread functions to the rectangular shape of the pixels of the line-scan camera and to improve the pixel sampling rates. An experimental SD-OCT is built to test and compare the performance of the *k*-space spectrometer with that of a conventional one. Design results demonstrate that this *k*-space spectrometer can reduce the nonlinearity error in *k-*space from 14.86% to 0.47% (by approximately 30 times) compared to the conventional spectrometer. The 95% confidence interval for RMS diameters is 5.48 ± 1.76 μm—significantly smaller than both the pixel size (14 μm × 28 μm) and the Airy disc (25.82 μm in diameter, calculated at the wavenumber of 7.548 μm^−1^). Test results demonstrate that the fall-off curve from the *k*-space spectrometer exhibits much less decay (maximum as −5.20 dB) than the conventional spectrometer (maximum as –16.84 dB) over the whole imaging depth (2.2 mm).

Spectral domain optical coherence tomography (SD-OCT) enables high-speed volumetric biomedical imaging with micrometric resolution and millimetric depth for scientific research and clinical study^1^,^2^,^3^. In SD-OCT, signal sensitivity tends to be weaker in deeper imaging regions. This depth-dependent loss in signal sensitivity is called “fall-off”^4^. Reducing sensitivity fall-off is a primary concern for the design of the spectrometer in a SD-OCT system.

In SD-OCT, depth profiles are constructed by the inverse Fourier transform (FT^−1^) of the interferograms under the premise that the spectrum is linearly sampled in wavenumber (*k*) space. However, the diffractive grating used in a conventional spectrometer disperses the light spectrum at the angles evenly spread versus wavelength (*λ*) rather than wavenumber (*k*). Digital rescaling of the spectrum from *λ*-space to *k*-space is required prior to FT^−1^, resulting in the fact that the spectral bands integrated by the camera pixels are unequal and leading to the fact that the signal sensitivity is decreased in depth^5^,^6^,^7^.

Ideally, increasing the number of pixels of the detector can improve the spectral-sampling frequency and reduce the depth-dependent sensitivity loss^8^,^9^. In practice, there is often a trade-off between the pixel number and the pixel size under a limited pixel array dimension. As a consequence, smaller pixel pitch often requires higher optics performance^10^. If the point spread functions (PSFs) for the dispersed spectrum are much larger than the pixel pitches (for example, more than two pixels are required to sample one PSF of a particular wavenumber), the optical performance of the spectrometer rather than the number of the pixels becomes the limit for the effective spectral sampling. In addition, the linear camera with more pixels tends to be more expensive, and both the time needed to capture an OCT frame and the time to process it is increased with more pixels.

An alternative method to reduce the sensitivity fall-off generated by the unequal sampling is to use the *k*-space spectrometer which disperses the spectrum optically with the necessary degree of equidistance in wavenumber. One of the possibilities to design such a spectrometer is to use a combination of a diffractive grating and a prism, where the nonlinearity in wavenumber caused by one can be offset by the other^11^. This combination can be either cemented as a “grism”^11^ or separated to provide more degrees of freedom in optimization^6^,^7^. By utilizing the *k*-space spectrometer in SD-OCT, previous literatures^6^,^7^ have demonstrated obvious improvement in sensitivity as well as reduction in computing time. However, these *k*-space spectrometers were designed for SD-OCT systems with limited axial resolution. In ref. 6, the center wavelength is 1310 nm and the bandwidth is 68 nm, corresponding to an axial resolution of 11.14 μm in air (8.07 μm in tissue, assuming an average refractive index 1.38); In ref. 7, two systems were designed. One has a center wavelength 1270 nm with a bandwidth of 70 nm, corresponding to an axial resolution of 10.17 μm in air (7.37 μm in tissue) and the other has a center wavelength 830 nm with a bandwidth of 40 nm, corresponding to an axial resolution of 7.60 μm in air (5.51 μm in tissue).

There is an increasing demand for developing the broadband or ultra-broadband OCT system with higher axial resolution to distinguish smaller structures, such as cellular boundaries and types^12^. This demand casts more difficulties for the *k*-space spectrometer design in the nonlinearity error correction and in the optical path difference (OPD) minimization for the dispersion element, as well as in the aberration correction for the focusing lens. Here we present a new design for the *k*-space spectrometer which outperforms the previous ones in a SD-OCT system with a center wavelength 840 nm and a bandwidth 160 nm, corresponding to an axial resolution of 1.95 μm in air (1.41 μm in tissue).

## Linear-in-*k* Optimization

In many biomedical applications, ultrahigh axial resolution is required for OCT systems to distinguish cellular boundaries and types^12^. Typically, an ultra-broadband light source is required to meet this purpose. We use a combined superluminescent laser diode (SLD) as the light source with a center wavelength of 840 nm and a bandwidth of 160 nm (760 nm–920 nm). Within this bandwidth (8.267 μm^−1^–6.830 μm^−1^ in *k*-space), 11 wavenumbers (*k*_*1*_ – *k*_*11*_) are chosen with an equal increment −0.144 μm^−1^ for calculation and design purposes. The center wavenumber *k*_*6*_ is 7.548 μm^−1^, corresponding to 832.38 nm in wavelength. The selected wavenumbers (*k*_*1*_ – *k*_*11*_) and their corresponding wavelengths (*λ*_*1*_ – *λ*_*11*_) are listed in Table 1.

Diffraction grating (e.g. transmissive volume phase holographic grating) is widely used as the dispersion component. If the collimated light is incident under a blaze condition for *λ*_*6*_ (*k*_*6*_), the blaze angle (in air) can be calculated as


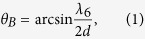


where *d* is the line spacing (*2d* for the period). The diffraction angle *α*_*i*_ (in air) for the corresponding wavelength *λ*_*i*_is given by


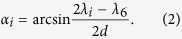


**Figure 1:**
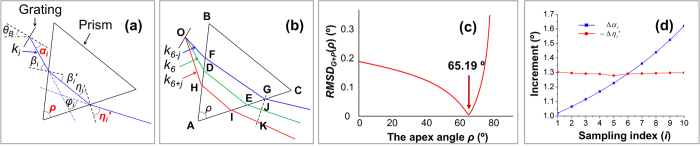
Linear-in-*k* optimization by combining a diffraction grating with an isosceles prism as the dispersion component in the SD-OCT spectrometer. (**a**) Chief ray tracing of an arbitrary light with the wavenumber of *k*_*i*_. *ρ* is the apex angle of the isosceles prism. *θ*_*B*_ is the Blaze angle (in air), *α*_*i*_ is the diffraction angle by grating (in air), *β*_*i*_ and *β*_*i*_′ are the incident and the refraction angles at the front surface of the prism, *η*_*i*_ and *η*_*i*_′ are the incident and refraction angles at the back surface of the prism, *ϕ*_*i*_ is defined as the deviation angle between the exit light and the entrance light. (**b**) Optical path lengths (*OPLs*) for the chief rays with the wavenumbers of *k*_*6−j*_, *k*_*6*_ and *k*_*6*+*j*_. (**c**) Minimization of the non-linearity error in *k*-space using root-mean-square deviation function (see eq. (8)) in the dispersion group comprised of a grating and a prism. (**d**) Demonstration of the significant improvement in linear dispersion angle distribution in *k*-space, by comparing the absolute increment of Δ*α*_*i*_for grating only and −Δ*η*_*i*_′ for grating-prism pair respectively.

From eq. (2), it is noted that *α*_*i*_is linear in wavelength (*λ*) rather than in wavenumber (*k*). To address this problem, we can use an isosceles prism (with the apex angle of *ρ*) to be paired with the grating as the dispersion group, as shown in Fig. 1(a,b).

In Fig. 1(a), an arbitrary light with the wavenumber of *k*_*i*_ coming out of the grating at the diffraction angle of *α*_*i*_, passes through the prism after two consecutive refractions. The incident and the refraction angles at the front surface are *β*_*i*_ and *β*_*i*_′ respectively. The incident and refraction angles at the back surface are *η*_*i*_ and *η*_*i*_′ respectively, where


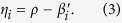


From Snell’s law, we have


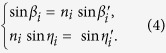


where *n*_*i*_ is the refractive index of the material related to the wavelengths (or wavenumbers). The prism material is Bk7, and the refractive indices *n*_*1*_ – *n*_*11*_ for different wavenumbers^13^ are listed in Table 2.

*ϕ*_*i*_ is defined as the deviation angle between the exit light and the entrance light, where


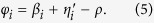


Therefore, *ϕ*_*i*_ is a function of the incidence angle *β*_*i*_, the apex angle of the prism *ρ*, and the refractive index *n*_*i*_ from eqs (3,4,5).

For the reference wavenumber *k*_*6*_ (7.548 μm^−1^), we have


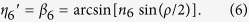


For other wavenumbers, *β*_*i*_ can be expressed as


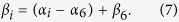


From eqs (3–4, 6–7), *η*_*i*_′ can be calculated accordingly, and it is a function of *α*_*i*_ and *ρ*.

For a grating with 1200 lines/mm (*d* = 0.83 μm), *θ*_*B*_ is calculated as 29.96° according to eq. (1). *α*_*1*_ – *α*_*11*_ are calculated based on eq. (2) and are listed in Table 2.

To optimize the linearity of the dispersion spectrum in *k* space, we set a root-mean-square deviation function *RMSD*_*G*_ _+_ _*P*_(*ρ*) for the grating-prism (G + P) group as


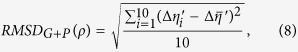


where


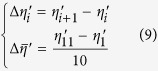


Based on eqs (8, 9), *ρ* is optimized in the angular range of 0°–90° and the optimized value is 65.19°, where *RMSD*_*G*_ + _*P*_(*ρ*) has the minimum value of 0.006°, as shown in Fig. 1(c). Under this situation, *β*_*1*_– *β*_*11*_*, β*_*1*_′ – *β*_*11*_′*, η*_*1*_ – *η*_*11*_and *η*_*1*_′ – *η*_*11*_′ can be all calculated (Table 2). The total dispersion angle range (*η*_*11*_′−*η*_*1*_′) is −12.93° with the average increment of −1.29°.

Figure 1(d) compares the increments of Δ*α*_*i*_(for grating only) and −Δ*η*_*i*_′ (for the grating-prism pair) respectively. It is obvious that significant improvement of linearity in dispersion as a function of wavenumber has been achieved after pairing the grating with prism. We can also calculate the coefficients of variation in linearity (CVLs) for the output angles from the grating only (*α*_*1*_ – *α*_*11*_) and from grating + prism (*η*_*1*_′ – *η*_*11*_′) in *k*-space as


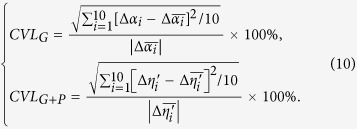


Quantitative calculations based on eq. (10) demonstrate that by utilizing grating and prism together as the dispersion group in our new design, the *k*-space angular nonlinearity errors can be greatly reduced from 14.86% (*CVL*_*G*_) to 0.47% (*CVL*_*G*_ _+_ _*P*_). In other words, we can achieve constant dispersion as a function of wavenumber with the linearity up to 99.53% in *k*-space spectrometer over the wave bandwidth of 160 nm, from 760 nm to 920 nm.

## Optical Path Difference Reduction

As shown in Fig. 1(b), the grating disperses the light at the point of *O*. For the reference light with the wavenumber *k*_*6*_, its chief ray goes through the prism (shown as the triangle *ABC*) with the interaction points of *D* and *E. D* is the center point of 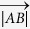, so 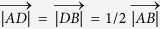; similarly, *E* is the center point of 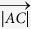, i.e., 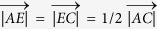, thus 
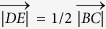
. If a 2-inch prism (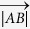 = 50.80 mm) is chosen, 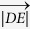 = 27.366 mm.

Given a pair of light rays with their wavenumbers of *k*_*6−j*_ and *k*_*6*_ _+_ _*j*_, which are centered at the reference wavenumber of *k*_*6*_ (*j* = 1, 2, …, 5), the chief ray of *k*_*6−j*_ goes through the prism with the interaction points of *F* and *G*, while the chief ray of *k*_*6*_ _+_ _*j*_ goes through the prism with the interaction points of *H* and *I*. To calculate the optical path difference (*OPD*) between *k*_*6−j*_ and *k*_*6*_ _+_ _*j*_, we set a reference line of *GK*, which is perpendicular to the output chief ray of *k*_*6*_ at point *J* and interacts with the output chief ray of *k*_*6*_ _+_ _*j*_ at point *K*.

Thus, the optical path difference (*OPD*) between *k*_*6*_ _+_ _*j*_ and *k*_*6−j*_ can be expressed as


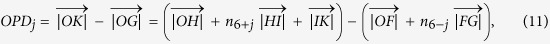


where 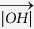, 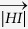, 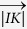, 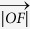 and 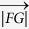 can be all related to the values of 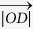 and 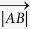. Thus eq. (11) can be modified as


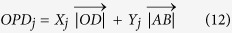


where


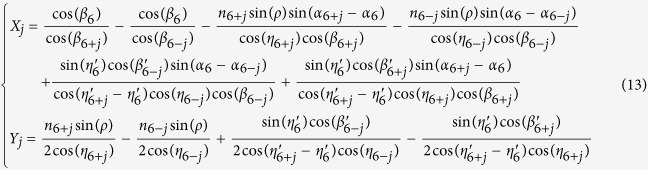


Substituting the values from Table 2 into eq. (13), we can calculate *X*_*j*_ and *Y*_*j*_ (j = 1, 2, …, 5) as shown in Table 3.

Under a given value of 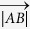, a corresponding value of 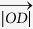 can be chosen to cancel the *OPD* between any pair of light rays with the wavenumbers of *k*_*6−j*_ and *k*_*6−j*_. However, all the *OPDs* between all pairs of the rays cannot be canceled simultaneously. If the average value of 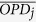 is canceled, where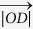 is calculated as 14.872 mm, the residual *OPDs* are: *OPD*(*k*_*7*_*-k*_*5*_) = −0.013 mm; *OPD*(*k*_*8*_*-k*_*4*_) = −0.022 mm; *OPD*(*k*_*9*_*-k*_*3*_) = −0.020 mm; *OPD*(*k*_*10*_*-k*_*2*_) = −0.002 mm; *OPD*(*k*_*11*_*-k*_*1*_) = 0.052 mm.

## Spectrometer Design Results

Zemax software (Zemax, LLC) was used to design and demonstrate this *k*-space spectrometer. The light coming out of the fiber (NA = 0.13) is first collimated by a 90° off-axis reflective parabolic mirror with the reflective focal length of 38.10 mm, then is dispersed by the grating and prism with the linear spectral distribution in *k*-space, and is finally focused by the focusing group onto the linear camera.

**Figure 2:**
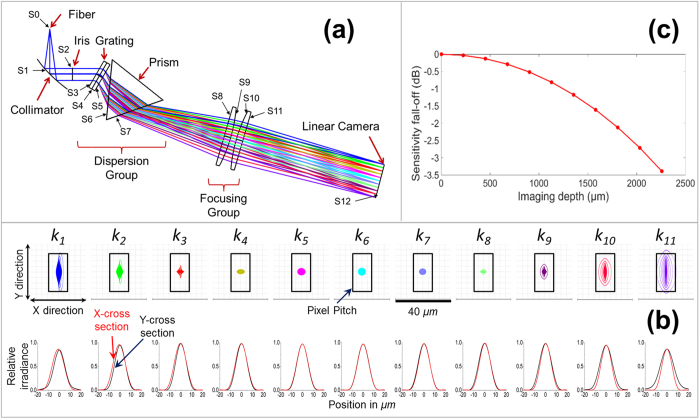
Optical design for the *k*-space spectrometer. (**a**) The *k*-space spectrometer is comprised of a collimator, a dispersion group (grating and prism), a focusing group and a linear camera. S0−S12 indicate the surface numbers. (**b**) Point spread functions (PSFs) for the wavenumbers of *k*_*1*_−*k*_*11*_ are narrower in the X direction than in the Y direction. The figures at the top show the spot diagrams (95% confidence interval for RMS diameters: 5.48 ± 1.76 μm). The black rectangles demonstrate the pixel shape/pitch (14 μm × 28 μm). The figures at the bottom are the cross section of the PSF profile, where the red lines show the cross section in the X direction and the black lines show the cross section in the Y direction. (**c**) Theoretical sensitivity fall-off calculation based on the average RMS spot diameter of 5.48 μm. The maximum fall-off is −3.39 dB at the imaging depth of 2.26 mm.

The linear camera has 2048 pixels and 14 μm × 28 μm pixel size. The required focal length for the focusing group is


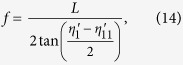


where *L* is the length of the effective pixel array of the line camera, equaling 28.67 mm. *f* is calculated to be 126.51 mm. To correct the aberrations (especially the field curves) for the dispersed spectrum, we can combine a commercial plano-convex lens (La1301-b, Thorlabs) with a custom designed aspherical lens. Their curved surfaces are facing each other. The final design results are shown in Fig. 2(a) and Table 4.

The image quality for *k*_*1*_– *k*_*11*_ can be evaluated by their point spread functions (PSFs) as shown in Fig. 2(b). The top row shows the spot diagrams referenced to the pixel size (14 μm × 28 μm) while the bottom row shows the cross-sectional PSFs in both X direction and Y direction. The spots are designed to be narrower in X direction than in Y direction to fit the pixel shape and to improve the sampling rate. The RMS diameters are 9.63 μm, 5.50 μm, 3.41 μm, 3.60 μm, 4.27 μm, 4.33 μm, 3.65 μm, 2.76 μm, 3.76 μm, 7.21 μm, and 12.13 μm respectively. The 95% confidence interval for RMS diameters are 5.48 ± 1.76 μm. All of the RMS diameters for *k*_*1*_– *k*_*11*_ are smaller than the Airy disc diameter of 25.82 μm (calculated at *k6*).

The sensitivity fall-off can be calculated theoretically based on eq. (15) consisting of a logarithm of the product of a sinc function and a Gaussian function, which are related to the Fourier transform of the shape of CCD pixels and the Gaussian beam profile in the spectrometer respectively^5^,^10^,^14^,^15^,^16^:


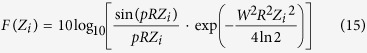


where *p* is the pixel size in *x* direction (*p* = 14 μm), and *R* is the reciprocal linear dispersion, indicating the width of the spectrum (in wavenumber) spread over 1 μm at the focal plane. *R* = Δ*k*/*p*, where Δ*k* is the wavenumber segment covered by one pixel. In our case, *R* = 5.015 × 10^–5^ μm^−2^. *Z*_*i*_ is the imaging depth and is in the range of 0–*Z*_*max*_, where *Z*_*max*_ is the maximum ranging depth (calculated in air). 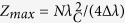, where *Δλ* is the bandwidth, and *N* is the number of pixels of the linear camera. Here, *Z*_*max*_is equal to 2.26 mm. *W* is the RMS spot diameter (i.e. 5.48 μm, from the simulation result). The theoretical sensitivity fall-off estimation is shown in Fig. 2(c), where the maximum fall-off value is −3.39 dB at 2.26 mm depth.

## Experimental Verification

**Figure 3:**
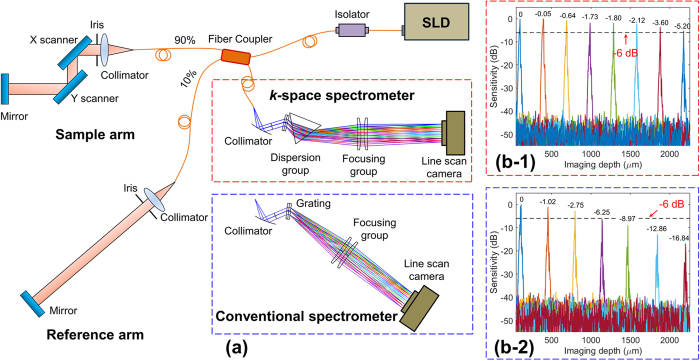
Detection sensitivity comparison between the *k*-space and the conventional spectrometers. An experimental SD-OCT set-up with both spectrometers is demonstrated in (**a**). SLD is a superluminescent diode with the bandwidth of 760 nm – 920 nm. The measurement results demonstrate obvious improvement in detection sensitivity in deeper imaging regions using the *k*-space spectrometer (**b-1**) in comparison with the conventional spectrometer (**b-2**).

We built an experimental SD-OCT system to compare the performance of this *k*-space spectrometer with a conventional spectrometer (with identical elements except the prism), as shown in Fig. 3(a). The OCT light source is a customized superluminescent diode (SLD, Inphenix, Inc.) with the central wavelength of 840 nm and the bandwidth of 160 nm (760 nm–920 nm). The maximum output power is ~7 mw. The light coming out of the SLD went through an isolator and then was split by a 90:10 fiber coupler (AC Photonics) into both the sample and reference arms. A line camera with 2048 pixels (Ev71yem4cl2014-ba9, E2v) was used in both spectrometers. The axial resolution is 1.95 μm in air. The maximum imaging depth is 2.26 mm (in air).

Two identical collimators (F280apc-780, Thorlabs) and mirrors were used in the sample and reference arms. The reference mirror was moved to induce optical path length differences (corresponding to the acquired imaging depths) between these two arms. Since the axial resolution and the sensitivity fall-off are irrelevant to the beam size, we controlled the irises (Sm1d12d, Thorlabs) in both arms to maximize the interference signal and to avoid camera saturation. The raw interference spectrum generated from each test location was acquired either by the *k*-space spectrometer or by the conventional spectrometer at a rate of 70 kHz through a camera link frame grabber (PCIE-1429, NI) into computer and then was processed via FFT to acquire the A-line intensity signal using the code in C++.

Depth related sensitivity measurements were compared between the *k*-space and conventional spectrometers in Fig. 3(b-1) and Fig. 3(b-2) respectively. It is noted that the fall-off curve from the *k*-space spectrometer measurement exhibits much less decay than the conventional spectrometer over the whole imaging depth. The maximum fall-off from the *k*-space spectrometer is −5.20 dB at 2.18 mm—smaller than the −6 dB criterion^8^,^15^. The maximum fall-off from the conventional spectrometer is −16.84 dB at 2.20 mm and the −6 dB fall-off position is ~1.10 mm. This comparison experiment demonstrates that the *k*-space spectrometer can effectively improve the signal sensitivity and image contrast in deeper imaging regions for SD-OCT system.

## Conclusion and Discussion

We have demonstrated a detailed theory for optimizing the dispersion components that consist of a diffraction grating and a prism. This method offers significant improvement in system sensitivity and it can be a guide for the designers in the OCT field. The key strategies include: (1) *k*-space linearity optimization by addressing the apex angle in the prism; and (2) optical path difference reduction among the dispersed spectrum for aberration minimization. By utilizing the grating and prism together as the dispersion group, we can significantly reduce the *k*-space angular nonlinearity errors from 14.86% to 0.47%. In other words, we can achieve constant wave-number dispersion with the linearity up to 99.53%.

We have designed a *k*-space spectrometer using Zemax. The RMS spot diameters for different wave numbers are in the range of 2.76 μm–12.13 μm with the 95% confidence interval of 5.48 ± 1.76 μm—much smaller than the Airy disc diameter of 25.82 μm (calculated at *k*_*6*_). In addition, the PSFs spread more in Y direction than in X direction in order to fit the pixel shape (28 μm in Y direction and 14 μm in X direction) of the line camera and to improve the sampling rates.

Comparison between the calculated [Fig. 2(c)] and the measured [Fig. 3(b-1)] sensitivity envelopes for the *k*-space spectrometer shows that the maximum drop values are −3.39 dB (at 2.26 mm) from calculation and −5.20 dB (at 2.18 mm) from measurement, respectively. This difference might be induced by the factors such as the spectrum shape of the light source, the quantum efficiency of the camera, manufacture and alignment errors, etc.

Comparison between the measured sensitivity envelopes in the *k*-space spectrometer [Fig. 3(b-1)] and the conventional spectrometer [Fig. 3(b-2)] shows that the sensitivity curve of the *k*-space spectrometer has much smaller decay over the whole imaging depth. The all-depth sensitivity fall-off for the *k*-space spectrometer is less than −6 dB criterion, while the −6 dB fall-off position for the conventional spectrometer is ~1.10 mm. It has been validated that the k-space spectrometer can effectively improve the signal sensitivity and image contrast in deeper imaging regions for SD-OCT system. In the next step, we will apply our system to imaging of the eye and other biomedical samples and the corresponding results will be reported in the near future.

## Additional Information

**How to cite this article:** Lan, G. and Li, G. Design of a *k*-space spectrometer for ultra-broad waveband spectral domain optical coherence tomography. *Sci. Rep.* **7**, 42353; doi: 10.1038/srep42353 (2017).

**Publisher's note:** Springer Nature remains neutral with regard to jurisdictional claims in published maps and institutional affiliations.

## Figures and Tables

**Table 1:** Selected wavenumbers (*k*_*1*_ – *k*_*11*_) and their corresponding wavelengths (*λ*_*1*_ – *λ*_*11*_).

Sampling index (*i*)	1	2	3	4	5	6*
**Wavenumber k**_**i**_**(μm**^**−1**^)	8.267	8.124	7.980	7.836	7.692	7.548
**Wavelength λ**_**i**_ **(nm)**	760.00	773.45	787.39	801.83	816.82	832.38
**Sampling index (*****i***)	**7**	**8**	**9**	**10**	**11**	
**Wavenumber k**_**i**_**(μm**^**−1**^)	7.405	7.261	7.117	6.973	6.830	
**Wavelength λ**_**i**_ **(nm)**	848.54	865.35	882.83	901.03	920.00	

^*^Referenced center wavenumber.

**Table 2:** Ray tracing values for the wavenumbers of *k*_*1*_ – *k*_*11*_ in the dispersion group.

*k*_*i*_	*k*_*1*_	*k*_*2*_	*k*_*3*_	*k*_*4*_	*k*_*5*_	*k*_6_*
***n***_***i***_	1.5116	1.5113	1.5110	1.5107	1.5104	1.5102
***α***_***i***_	24.37°	25.39°	26.45°	27.57°	28.73°	29.96°
***β***_***i***_	48.85°	49.87°	50.93°	52.05°	53.22°	54.44°
***β***_***i***_′	29.88°	30.39°	30.92°	31.46°	32.02°	32.60°
***η***_***i***_	35.31°	34.80°	34.27°	33.73°	33.17°	32.60°
***η***_***i***_′	60.90°	59.60°	58.30°	57.01°	55.72°	54.44°
***i***	***k***_***7***_	***k***_***8***_	***k***_***9***_	***k***_***10***_	***k***_***11***_	
***n***_***i***_	1.5099	1.5096	1.5093	1.5090	1.5087	
***α***_***i***_	31.25°	32.61°	34.05°	35.58°	37.20°	
***β***_***i***_	55.73°	57.10°	58.53°	60.06°	61.68°	
***β***_***i***_′	33.19°	33.79°	34.41°	35.05°	35.70°	
***η***_***i***_	32.00°	31.40°	30.78°	30.14°	29.49°	
***η***_***i***_′	53.15°	51.86°	50.56°	49.27°	47.97°	

^*^Reference.

**Table 3:** The values of *X*_*j*_ and *Y*_*j*_ for OPD minimization.

*j*	1	2	3	4	5
*X*_*j*_	5.386E-05	4.123E-04	1.407E-03	3.426E-03	6.959E-03
*Y*_*j*_	−2.643E-04	−5.562E-04	−7.958E-04	−9.571E-04	−1.015E-03

**Table 4:**
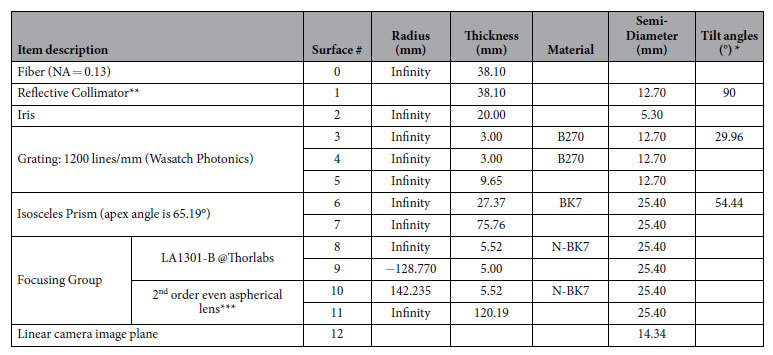
General design parameters for the *k*-linear spectrometer.

^*^Calculated on the chief ray for the central wavenumber (7.548 μm^−1^). Detailed tilted angles for all other wavenumbers are listed in Table 2.

^**^90° off-axis reflective parabolic mirror with silver coating. The reflective focal length is 38.1mm and the parent focal length is 19.05 mm.

^***^The 2^nd^ order term for this even aspherical surface is −4.268E-004.
